# Construction of antimicrobial peptide-drug combination networks from scientific literature based on a semi-automated curation workflow

**DOI:** 10.1093/database/baw143

**Published:** 2016-12-26

**Authors:** Paula Jorge, Martín Pérez-Pérez, Gael Pérez Rodríguez, Florentino Fdez-Riverola, Maria Olívia Pereira, Anália Lourenço

**Affiliations:** 1 CEB - Centre of Biological Engineering LIBRO - Laboratory of Research in Biofilms Rosário Oliveira, University of Minho, Braga, Portugal; 2 ESEI - Department of Computer Science, University of Vigo, Ourense, Spain; 3 CEB - Centre of Biological Engineering, University of Minho, Braga, Portugal

## Abstract

Considerable research efforts are being invested in the development of novel antimicrobial therapies effective against the growing number of multi-drug resistant pathogens. Notably, the combination of different agents is increasingly explored as means to exploit and improve individual agent actions while minimizing microorganism resistance. Although there are several databases on antimicrobial agents, scientific literature is the primary source of information on experimental antimicrobial combination testing. This work presents a semi-automated database curation workflow that supports the mining of scientific literature and enables the reconstruction of recently documented antimicrobial combinations. Currently, the database contains data on antimicrobial combinations that have been experimentally tested against *Pseudomonas aeruginosa*, *Staphylococcus aureus*, *Escherichia coli*, *Listeria monocytogenes* and *Candida albicans*, which are prominent pathogenic organisms and are well-known for their wide and growing resistance to conventional antimicrobials. Researchers are able to explore the experimental results for a single organism or across organisms. Likewise, researchers may look into indirect network associations and identify new potential combinations to be tested. The database is available without charges.

**Database URL:** http://sing.ei.uvigo.es/antimicrobialCombination/

## Introduction

Antimicrobial resistance is currently one of the major health threats worldwide. Recent statistics from the Centers of Disease Control and Prevention (CDC) indicate that, each year, at least 2 million people become infected with antibiotic-resistant bacteria in the USA and 23 000 people die as a direct result of these infections (http://www.cdc.gov/drugresistance/). Other reports state over 700 000 deaths per year worldwide (1).

Antimicrobial agents, i.e. antibiotics and similar drugs, have been so widely overused and misused that the infectious organisms were selectively pressured to develop resistance towards them (2). The main mechanisms of action of antimicrobials include interference with cell wall synthesis (e.g. beta-lactams), inhibition of protein synthesis (e.g. tetracyclines), interference with nucleic acid synthesis (e.g. fluoroquinolones and rifampin), inhibition of a metabolic pathway (e.g. trimethoprim-sulfamethoxazole), and disruption of bacterial membrane structure (e.g. polymyxins and daptomycin) (3). Microorganisms may be intrinsically resistant to one or more classes of antimicrobial agents, or may acquire resistance by *de novo* mutation or via the acquisition of resistance genes from other organisms. Acquired resistance genes may enable the microorganism to produce enzymes that destroy the antimicrobial drug, to express efflux systems that prevent the drug from reaching its intracellular target, to modify the drug’s target site, or to produce an alternative metabolic pathway that bypasses the action of the drug. The number of multi-drug resistant (MDR) strains and pandrug resistant isolates is growing continuously and rendering conventional antibiotics less effective (4, 5).

Clinical and microbiological research is thus devoting significant attention to the understanding of antimicrobial resistance phenomena, the discovery of alternative agents (or mechanisms of action), and the development of new antimicrobial strategies (6, 7). In this context, antimicrobial peptides (AMP) are recognized as a promising antimicrobial agents that have a broad spectrum of activity and show low specificity in terms of molecular targets, which helps lower the chance of microorganisms developing resistance (8). AMP support antimicrobial action by aiding cellular processes like cytokine release, chemotaxis, antigen presentation, angiogenesis and wound healing (9, 10), and are active against biofilms, which are one of the most concerning mechanisms of microbial resistance and a major cause of resilient infections, such as biomaterial related infections and chronic infections (11–13).

Now, alongside the discovery of new antimicrobial agents, researchers are looking into potentiating the action of both old and new substances. In particular, one possible solution is to look for synergic combinations of two or more antimicrobial agents, which increase the antimicrobial spectrum and potentiate the individual efficacy of the agents, while avoiding antimicrobial resistance and reducing toxicity and other side effects (6). The challenge resides in the rational combination of compounds and in finding the most promising mechanisms of action to treat particular infections and to overcome specific mechanisms of resistance.

The huge number of compounds available and the variety of possible combinations is leading to the accumulation of a large and highly diversified volume of experimental data. Several public databases store information on drugs, AMP and other compounds with antimicrobial potential, but scientific literature remains as the primary source of information (14–18). Databases do not provide enough details on susceptibility testing that may be used by researchers to evaluate individual and joint antimicrobial effects.

Within this scope, mining the bibliome for experimentally validated antimicrobial combinations has the potential to provide researchers insights on existing results and infer the most promising combinations to be tested next. Previous works have successfully developed text mining methods and tools for the reconstruction of pharmacokinetic experimental evidence (19), adverse drug-drug interactions (20), and drug-gene and drug-disease interactions (21, 22), among others. Although the focus of these works is different, the extraction of experimental evidence of antimicrobial agent combinations can get inspiration from these computational workflows and use some of the tools and resources.

Therefore, this work presents a semi-automated knowledge extraction workflow that was developed to allow the extraction of correlative relationships about the combination of antimicrobial agents from scientific literature. This workflow integrates state-of-the-art text mining technologies and expert manual curation in support of the compilation of detailed information on antimicrobial combinations (involving both drugs and AMP) tested against major pathogenic bacteria and fungi. Moreover, it resorts to network representation as means to enable query and visualization at large scale and help users explore direct and indirect associations in an easy and comprehensible manner.

Current, the database contains 1556 combinations, encompassing 345 AMP and 282 drugs, tested on *P. aeruginosa*, *S. aureus*, *E. coli*, *L. monocytogenes* and *C. albicans*. Presently, and to the best knowledge of the authors, no other database provides information regarding the testing of AMP-related combinations. This database is publicly available at http://sing.ei.uvigo.es/antimicrobialCombination/.

## Materials and Methods

This section describes the integrated and semi-automated data curation workflow developed to reconstruct experimentally validated AMP and drug combinations. This curation workflow is iterative, i.e. the aim is to keep up with new findings about antimicrobial combinations and therefore, future versions of the database will likely cover new antimicrobial agents as well as a broader scope of pathogenic microorganisms. Accordingly, the workflow is designed to enable domain-specific curation with active lexicon enrichment and calibration of automatic procedures.

**Figure 1.**
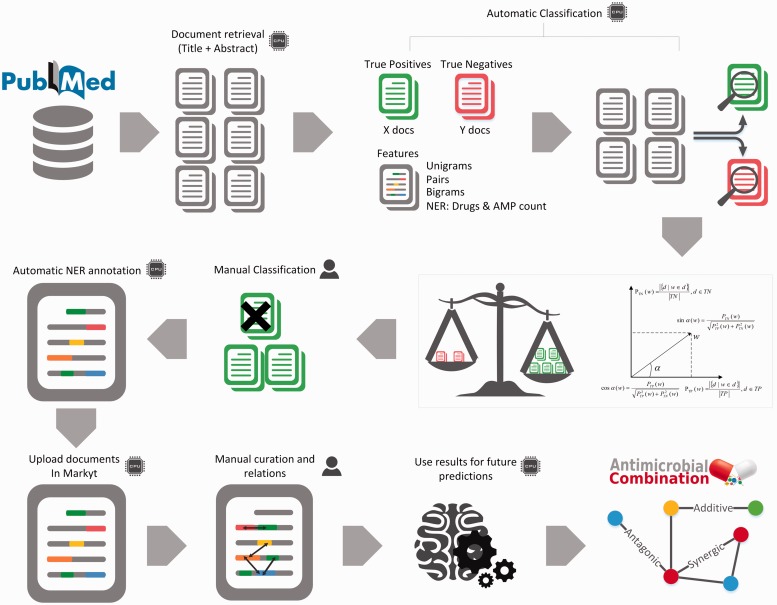
Schema of the integrated and semi-automated curation workflow. The curation process starts with the automatic search and retrieval of PubMed records. Text mining methods support the annotation of relevant entities (antimicrobial agents, methods for antimicrobial susceptibility testing, type of agent combination, and organisms) and the assessment of document relevance. Articles deemed relevant are further curated manually by experts that revise automatic annotations and look into additional information about the types of combinations and other relevant details of the experiments. Articles describing previously uncharacterized AMP combinations are added to the network.

As illustrated in Figure 1, the developed workflow integrates modules for the retrieval of target documents, the processing, annotation and analysis of their contents, and the visualization of the combination profiles of antimicrobial agents. A prototype of this data curation workflow was previously implemented for an initial reconstruction of antimicrobial combinations tested against *P. aeruginosa* (23). The lessons learned about how to integrate the automatic and manual processes of curation, and on how to apply such curation to other organisms and specific antimicrobials, reflected directly in the architecture of the workflow presented here.

Next, we detail the main aspects of the current workflow.

### Document retrieval and pre-processing

The NCBI (National Center for Biotechnology Information) Entrez Utilities Web services are used to access the PubMed library, search for potentially relevant articles and download the publication details, including the titles and abstracts to be further processed (24).

The aim here is to find scientific literature about the experimental validation of antimicrobial combinations, namely combinations involving common, commercial drugs (e.g. antibiotics, disinfectants) and AMP (natural or designed). Therefore, the scope of the search is narrowed to documents whose title or abstract mentions terms that commonly denote the test of agent combinations (e.g. ‘combination’, ‘synerg*’ or ‘antagon*’, where the ‘*’ is a wildcard), and experimental methods specific to antimicrobial combination susceptibility testing (e.g. ‘checkerboard assay’ or ‘FBC’). Moreover, the search is organism-centric (i.e. we specify the organism) as we chose to compile a meaningful set of documents about a subset of organisms as opposed to have a set of documents that covers a wide range of organisms but is not able to provide a decent understanding on research outcomes for each organism. Most notably, the database covers studies on *P. aeruginosa*, *S. aureus*, *L. monocytogenes*, *E. coli* and *C. albicans*, which are major MDR pathogens and attract considerable attention from the research community.

Basic text processing steps, namely tokenization, stemming, and stop word removal, required by some of the entity recognition and document assessment algorithms, were implemented using Apache Lucene (http://lucene.apache.org/). These procedures are applied at document arrival and after combining title and abstract into a single text.

### Entity recognition

Named entity recognition methods are used to identify mentions of critical entities, notably antimicrobial agents, experimental methods specific to antimicrobial susceptibility testing, and organisms. These annotations are used to index document contents and reconstruct meaningful relations. Moreover, the number of unique drug and AMP mentions per abstract is also used as a classification feature by the document relevance model.

The automatic recognition of antibiotic and AMP textual mentions is accomplished with the help of in-house built dictionaries.

We have downloaded the drug lexicon from DrugBank and includes both FDA-approved drugs and experimental drugs (14). The peptide lexicon was downloaded from CAMP (17) and LAMP (25), focusing on peptides extracted from natural sources. Additional lexicon on potentially bioactive compounds and substances (e.g. enzymes, natural products and synthetic products) was retrieved from the databases CHEBI (18), PubChem (26), CHEMBL (27) and the protein catalogue of Uniprot (28). All this information was parsed and stored in a custom database and contains a total of 284 337 entries, including 280 503 drugs and 3749 drug-like bioactive compounds and peptides. The average length of common entity names is 30.27 and the average number of synonyms is 11.

The dictionary used in entity recognition represents a subset of these contents. Contents were filtered according to the role, action or classification associated to the agents by database curators. Our experts explored database-specific classification/annotation and provided a list of the filtering terms. The dictionary contains a total of 36 259 entries, including 32 772 drugs and 3487 drug-like bioactive compounds and peptides. Further information about the extraction, parsing and filtering steps of this data workflow can be found in Supplementary Materials.

Similarly, a dictionary-based approach is used to detect textual mentions of the methods used to test the efficacy of antimicrobial agent combinations, and the description of combination effects, such as synergies and antagonisms. This dictionary was built in-house in collaboration with field experts (29, 30) (see Supplementary Material 2). Finally, the state-of-the-art NER taggers LINNAEUS (31) and ABNER (32) are used to identify species and drug targets, respectively.

### Document relevance assessment

The *P. aeruginosa*, *S. aureus* and *E. coli* documents retrieved from PubMed were manually labelled by experts to train of the relevance assessment method. Initially, the title and abstract texts of these documents are stemmed with the Porter algorithm (33), filtering out short words with two or fewer letters, and removing common stop words. The predictive ability of remaining words is then examined. The probability p_TP_(*w*) that a word *w* appears in a positive abstract (i.e. relevant abstract) is calculated as the ratio of the number of positive abstracts containing *w* over the total number of positive abstracts. Similarly, the probability p_TN_(*w*) that a word *w* appears in a negative abstract (i.e. irrelevant abstract) is calculated as the ratio of the number of negative abstracts containing *w* over the total number of negative abstracts. Then, words are ranked according to the score:

$$S(w)=\left|p_{TP}\left(w\right)-\;p_{TN}(w)\right|$$

Words with the highest score S tend to be associated with either positive or negative abstracts and thus, are assumed to be good features for classification.

The predictive ability of pairs of words immediately adjacent in the text and of unique pairs of words that co-occur in the documents is also considered. These two additional feature sets are obtained from the stemmed word features in the first set. The predictive ability of such pairs of words (*w*_i_, *w*_j_) is calculated as the probability of appearing in a positive or negative abstract, p_TP_(*w*_i_, *w*_j_) and p_TN_(*w*_i_, *w*_j_), respectively.

In addition to unigrams, bigrams and co-occurring words features, the predictive model also takes into account the number of unique drug and AMP mentions per abstract *a*, nd(*a*) and namp(*a*), respectively (see details in ‘Entity recognition’ section).

The information from the various textual and entity count features is integrated in a variable trigonometric threshold linear classifier (34–36). Typically, this classifier defines a decision surface, i.e. a p_TP_/p_TN_ plane, where those feature terms with better predictive ability are close to either one of the axes. Any feature term *w* is a vector on this plane, and therefore term relevance to each of the classes can be measured with the traditional trigonometric measures of the angle α, of this vector with the p_TP_ axis. That is, the cos(α) is a measure of how strongly terms are exclusively associated with positive abstracts, and sin(α) is a measure of how strongly terms are exclusive of negative abstracts. So, for every abstract *a*, relevance is assessed on the basis of the sum of the contribution of all feature terms for a positive (P) and negative (N) decision:

$$\begin{array}{c} P(d)=\sum_{w\in d}\;\text{cos}\;(\alpha (w))=\sum_{w\in d}\frac{p_{P}(w)}{\sqrt{p_{P}^{2}(w)+p_{N}^{2}(w)}}\\ N(d)=\sum_{w\in d}\;\text{sin}\;(\alpha (w))=\sum_{w\in d}\frac{p_{N}(w)}{\sqrt{p_{P}^{2}(w)+p_{N}^{2}(w)}} \end{array}$$

The decision of whether a given abstract *a* is relevant (positive) or not (negative) is computed as follows:

$$\left(\frac{\text{nd}(a)+\text{namp}(a)}{\beta }\right)\geq \left(-\frac{P(a)}{N(a)}+\lambda \right)$$

here λ is a constant threshold for deciding whether an abstract is positive or negative. This threshold is subsequently adjusted for each abstract *a* with the factor $\left(\frac{nd\left(a\right)+namp(a)}{\beta }\right)$, where β is a constant, and $nd\left(a\right)+namp(a)$ is the number of unique drugs and AMP in the abstract as described in the feature selection subsection.

The values of λ and β are optimized by performing k-fold tests (*k* = 4) on the training data. Specifically, we swept the following parameter range: *λ* ∈ [0.25, 10] and *β* ∈ [1, 50], in steps of Δ*λ* = 0.25 and Δ*β* = 2. For each (*λ*; *β*) pair, we compute the mean of the F-score and accuracy measures for each of the four *k*-fold tests. We rank classifiers according to the mean value of *F*-score (*r*_F_) and accuracy (*r*_A_) and then, we rank all classifiers tested according to the rank product, i.e. $R=r_{F}\times r_{A}$. Supplementary Materials details this evaluation.

### Expert manual curation

The Markyt annotation tool (37) supports document manual curation and feeds several modules of the developed workflow as follows: provides insights on possible dictionary updates to the entity recognition module; makes available information on manual relevance assessment to enable the update of the automatic relevance assessment model; and, outputs the information necessary to reconstruct the combination networks, i.e. antimicrobial agents, the antimicrobial combinations and further details provided by the annotated textual evidences.

Manual curation guidelines address both relevance assessment and semantic annotation. A document is labelled as relevant if it describes the experimental testing of one or more antimicrobial combinations and at least one of these combinations involves an AMP. Moreover, curators were instructed to exclude non English documents and reviews.

The biological concepts considered important for the reconstruction of the AMP-drug combination networks are described in Table 1. Basically, interest is set on the identification of the antimicrobial agents tested, the infectious organism(s) targeted (including whenever possible the strain), the mode of growth of the microbial culture, and the antimicrobial susceptibility method(s) used.

**Table 1.** Relevant entities to the reconstruction of antimicrobial combination networks

Named entity	Annotation procedure	Description
**Antimicrobial agent**	Semi-automatic	Commercial drugs, antibiotics, antifungals, AMP (natural and designed), enzymes, disinfectants
**Organism**	Semi-automatic	Pathogenic bacteria and fungi
**Strain**	Manual	Reference strain, isolated strain (clinical isolate, food isolate)
**Mode of growth**	Manual	Planktonic, biofilm, *in vivo*
**Experimental tests**	Semi-automatic	Checkerboard assay and/or FIC and/or FBC determination
		Time-kill assay
		Other (e.g. MIC, MBC, bacterial counts, cell viability, etc)

From our experience, curators are usually able to check document relevance by analysing the abstract of the articles. However, it is often the case that full-text examination is required to confirm relevance and to extract some of the information of interest, notably details on the experimental procedures. In each iteration, experts revise the documents automatically labelled as relevant by the predictive model and a fraction of the documents labelled as irrelevant (with better ranking score). As such, we may identify deficiencies in automatic entity recognition (namely in dictionary coverage), and the necessity to recalibrate the λ and β thresholds of the predictive assessment model.

For the automatic annotation to be considered accurate, it should correctly identify the type of the entity and mark an acceptable fragment of the corresponding textual mention. Inconsistencies, glitches, misses, and interpretation issues are amended by the experts and duly documented for future improvement of the workflow (namely, to improve the vocabulary and matching rules supporting automatic annotation, and the priority given to NER tool outputs).

Abstracts mainly contain a summary of the obtained results, and in particular, they typically describe only the best performing combinations. Therefore, the full-texts of the documents deemed relevant are always manually curated in order to annotate all the combinations tested. The curators manually relate the antimicrobial agents forming each combination and classify the combinations based on the described effects. Four different types of combinations are considered: ‘synergic’, i.e. the combined action is superior to the sum of the isolated actions; ‘additive’, i.e. the combined action is equal to the sum of the isolated actions; ‘indifferent’, i.e. the combined action is equal to the action of the most active single agent; and, ‘antagonic’, i.e. the combined action is inferior to the action of the most active single agent. In some cases, other categories named ‘additive/indifferent’ or ‘synergic/additive’ are used to denote that results were not conclusive.

Although curating the full-texts, curators have to analyse the materials and methods and the results sections in order to understand the methodology applied in the study and the results obtained for each combination. If the typical methodologies for testing combinations *in vitro* are applied (e.g. checkerboard assay, fractional inhibition concentration (FIC) and/or FBC determination, and time-kill assay), the annotation of the results is easier, because the conclusions are usually quantifiable. For example, synergy can be indicated by FIC or FBC ≤ 0.5 or by log decrease ≥ 2 on viable cells when comparing the action of the combinations with the action of the most active single agent (38). However, the interpretation of results obtained by less standard methodologies may be more challenging. Likewise, many documents do not describe textually the results of all the tested combinations, and curators often need to analyse data shown in graphs or tables to be able to document all combinations and their results properly. This is particularly true for less successful combinations, i.e. non synergic combinations.

### Network visualization and search

The database of antimicrobial combinations is publicly accessible through a Web-based interface. This interface allows users to formulate queries at different levels of specificity, e.g. filtering the antimicrobial combinations by organism, antimicrobial agent and combination effect. Specifically, a Cytoscape Web (39) based interactive network browser supports user customized database queries and the visualization of network relationships between antimicrobial agents.

Network representation offers an intuitive and visually appealing means to observe and navigate a potentially large number of relationships. Furthermore, the analysis of network topology provides descriptive statistics about the agents and types of combinations matching the user query and enables the inference of indirect associations.

Network nodes denote antimicrobial agents and edges identify experimentally validated combinations among agents. Accordingly, node records describe the antimicrobial agents and cross-link with primary chemical databases, whereas edge records detail information of the experimental results (i.e. type of combination, strain, mode of growth and experimental methods). Moreover, both nodes and edges are linked to the supporting literature.

The size and the colour of the node are dependent on its degree, i.e. the number of antimicrobial combinations in which the agent participates, and the width of the edge indicates the number of documents that describe the combination. Additionally, the shortest path algorithm enables the identification of indirect relations between antimicrobial agents, i.e. the identification of combinations not yet tested, but apparently reasonably possible considering the documented results.

Users are able to navigate through tested combinations and identify which agents have already been tested together and those that have not been tested together but are recurrently tested with similar agents. Also, users may look into specific types of combinations, e.g. synergic effects, as well as look for combinations tested against a particular target or across multiple organisms.

Besides the Cytoscape Web browser, this Web interface utilizes consolidated Web technologies. PHP programming language (version 5.5) and the MySQL database engine (version 5.1.73) support the server side operations. HTML5 (http://www.w3.org/TR/html5/) and CSS3 technologies (http://www.css3.info/) provide for common interface features. Finally, Ajax and JQuery (http://jquery.com/) technologies help in user-system interaction, such as query facets.

## Results and discussion

### Database statistics

Currently, the database contains primarily data on antimicrobial combinations that have been experimentally tested against *P. aeruginosa*, *S. aureus*, *E. coli*, *L. monocytogenes* and *C. albicans*, which are prominent pathogenic organisms and are well-known for their wide and growing resistance to conventional antimicrobials.

The number of documents retrieved from the literature was far greater for the bacteria *S. aureus* and *E. coli* (more than a thousand documents each) than for *P. aeruginosa*, *L. monocytogenes* and *C. albicans* (a few hundred documents each) (Table 2). Interestingly, this difference is less noticeable when considering the number of documents deemed relevant. For example, the number of relevant documents for *E. coli* is approximately 1/20 of the number of retrieved documents and, in contrast, this same proportion is ∼1/5 for *L. monocytogenes*. Considering that *S. aureus* and *E. coli* are highly studied pathogens, it was expected that PubMed queries could yield large result sets with a considerable number of false positives. It was often the case that abstracts contained relevant entities to the target domain, but expert manual curation (sometimes resorting to full-text examination) determined that the textual context of these entities was not of interest. For example, the document with PMID: 18326181 refers to potentially relevant keywords, such as ‘nisin’, ‘combination effects’ and ‘*E.* *coli*’. However, experts determined that the AMP nisin was not tested in combination with any other drug/AMP against *E. coli*.

**Table 2.** General statistics about the retrieved and annotated documents per organism

Microorganism	Retrieved documents	Relevant documents	Annotated combinations	Download date
*P. aeruginosa*	574	109	658	June–July (2015)
*S. aureus*	1078	101	462	June–July (2015)
*E. coli*	1472	69	283	June–July (2015)
*L. monocytogenes*	190	34	56	July–August (2015)
*C. albicans*	373	29	97	September–October (2015)

The analysis of the annotated combinations provided some insights about the type of studies that are being performed and which AMP and drugs (and mechanisms of action) are being combined. As shown in Table 3, studies follow a similar path regardless the organism: AMP are in their majority combined with antibiotics and antifungals; combinations of only AMP represent only 1–19% of the total combinations tested. The only exception to this scenario is *L. monocytogenes*, for which no antibiotic or antifungal was used, and AMP were mostly combined with other agents such as plant extracts and various chemicals (i.e. acids, alcohols, salts, organic compounds); however, AMP-AMP combinations still represent a small percentage (17.86%) of the total number of combinations. The recycling and potentiation of old and current antibiotics with the aid of other antimicrobials or antimicrobial adjuvants is one of the current antimicrobial strategies to fight antimicrobial resistance (6) and can explain these percentages.

**Table 3.** Overview of the number of annotated antimicrobials and combinations

Microorganism	AMP combination with		Mode of growth		Experimental test		Combination type			
	AMP	Anti(B/F)	P	B	CKB and/or FIC/FBC	T-K	S	Ad	I	A
***P. aeruginosa***	59 (8.97%)	489 (74.3%)	628 (95.4%)	18 (2.73%)	399 (60.7%)	85 (12.9%)	317 (48.2%)	118 (19.6%)	164 (24.9%)	48 (7.29%)
***S. aureus***	47 (10.2%)	280 (60.6%)	398 (86.2%)	40 (8.66%)	259 (56.1%)	44 (9.52%)	238 (51.5%)	127 (27.5%)	62 (13.4%)	35 (7.58%)
***E. coli***	53 (18.7%)	105 (37.1%)	268 (94.7%)	6 (2.12%)	152 (53.7%)	18 (6.36%)	102 (36.0%)	68 (24.0%)	71 (25.1%)	42 (14.9%)
***L. monocytogenes***	10 (17.9%)	0	47 (83.9%)	0	12 (21.4%)	0	38 (67.9%)	5 (8.93%)	13 (23.2%)	0
***C. albicans***	1 (1.03%)	51 (52.6%)	96 (99.0%)	0	55 (56.7%)	3 (3.09%)	54 (55.7%)	10 (10.3%)	11 (11.3%)	22 (22.7%)

Legend: Anti(B/F), antibiotic and antifungal; P, planktonic; B, biofilm; CKB, checkerboard; T-K, time-kill; S, synergic; Ad, additive; I, indifferent; A, antagonic.

Note: Synergic/additive plus additive/indifferent, and indifferent/antagonic combinations are included in the Ad and I categories, respectively. The percentages are relative to the total number of combinations for each microorganism.

Another interesting observation is that AMP combinations are being tested mainly on planktonic cultures (84–99%) (Table 3). Today, it is well-known that most bacteria are naturally present in consortia, i.e. a biofilm mode of growth, and most infections, namely the most resilient, are related to these microbial consortia (40). Therefore, as it stands, current studies give limited information about the effect of the tested combinations in real life conditions and it would be desirable to have more experimental data on biofilms.

Finally, one may observe that the experimental methods most used in these studies are the checkerboard, the FIC determination and the fractional bactericidal concentration (FBC) determination (the latest two are usually coupled), and the majority of the combinations resulted in synergic outcomes (Table 3). Both findings were somewhat expected since the referred methods are standard for this type of combinatorial research and scientific articles often tend to report only/majorly positive outcomes.

Table 4 presents the top 3 most annotated AMP, drugs and organism strains. Regarding the most annotated AMP, it is interesting to note that some of them are tested across organisms, with a total of seven different AMP out of a possible 15 AMP (top 3 AMP * 5 different organisms). Polymyxins, specifically colistin (polymyxin E) and polymyxin B, are one of the most annotated AMP groups. Both AMP groups were present in the top most annotated AMP for *P. aeruginosa*, *L. monocytogenes* and *C. albicans*. Polymyxins are mainly active against Gram-negative pathogens, including very important nosocomial pathogens such as *E. coli* and *P. aeruginosa* (41). Nevertheless, colistin was also tested in combinations against the Gram-positive *L. monocytogenes* and the yeast *C. albicans* (Table 4), illustrating that researchers are taking advantage of its diverse spectrum of activity to tackle a broader set of infection agents.

**Table 4.** The top three most annotated AMP, drugs and organism strains in the database

Microorganism	AMP	Drug	Strain
***P. aeruginosa***	Colistin (10.9%)	Novobiocin (16.9%)	Clinical isolates (53.8%)
	Polymyxin B (6.69%)	Ciprofloxacin (6.38%)	ATCC 27853 (17.6%)
	Polymyxin B nonapeptide (5.78%)	Imipenem (4.41%)	PAO1 (13.3%)
***S. aureus***	Nisin (12.0%)	Vancomycin (8.43%)	Clinical isolates (20.1%)
	Lactoferricin B (10.0%)	Lysostaphin (4.82%)	ATCC 25923 (16.8%)
	CA-MA (3.73%)	Ciprofloxacin (4.82%)	ATCC 43300 (12.2%)
***E. coli***	Nisin (5.95%)	NaCl (6.52%)	ATCC 25922 (37.3%)
	Lactoferricin B (4.76%)	Lysozyme (5.22%)	Clinical isolates (11.8%)
	LL-37 (4.46%)	Lactoferrin (3.48%)	O157:H7 (7.14%)
***L. monocytogenes***	Nisin (68.2%)	NaCl (6.52%)	Scott A (24.0%)
	Pediocin PA-1 (4.55%)	LPS (6.52%)	ATCC 7644 (9.33%)
	Colistin (4.55%)	EDTA (6.52%)	ATCC 19113 (4.00%)
***C. albicans***	Lactoferricin B (11.2%)	Caspofungin (14.6%)	SC5314 (15.6%)
	Colistin (8.16%)	NaCl (10.4%)	Clinical isolates (15.6%)
	Polymyxin B (7.14%)	Fluconazole (9.38%)	ATCC 90028 (14.1%)

Note: The percentage is relative to the total number of annotations for the respective entity class.

Nisin and lactoferricin B are also commonly tested in three of the five microorganisms. Nisin is the main representative of the AMP class of lantibiotics and is commercially available as a food additive. This AMP is known to be active against major Gram-positive pathogens such as *L. monocytogenes* and *S. aureus* (42), but it is also being tested against the Gram-negative *E. coli* (Table 4). On the other hand, lactoferricin B is a naturally occurring AMP in mammals with various intracellular targets against bacteria, and has well documented action against major pathogens such as *E. coli* and *S. aureus* (43), and now it is also tested against yeast (Table 4).

Regarding the most combined drugs, traditional antibiotics, such as ciprofloxacin, are the most used in the combinations tested against *P. aeruginosa* and *S. aureus* (Table 4). Likewise, antifungals are commonly integrated in the combinations tested against *C. albicans* (Table 4). However, it is interesting to note that the substance most combined with AMP in *E. coli* and *L. monocytogenes* studies is NaCl (sodium chloride) (Table 4). Many AMP have reduced antimicrobial activity in mediums with high ionic strengths or even at physiological salt concentrations (44). Therefore, recent combination studies aim to understand this phenomenon.

Finally, a considerable number of the AMP combinations are tested against clinical isolates (strains isolated from real-life infections). These strains are the best representatives of the resistance encountered on infection scenarios and therefore, serve as a more realistic baseline of comparison with reference strains. In fact, studies usually cover more clinical isolates than reference strains.

**Figure 2.**
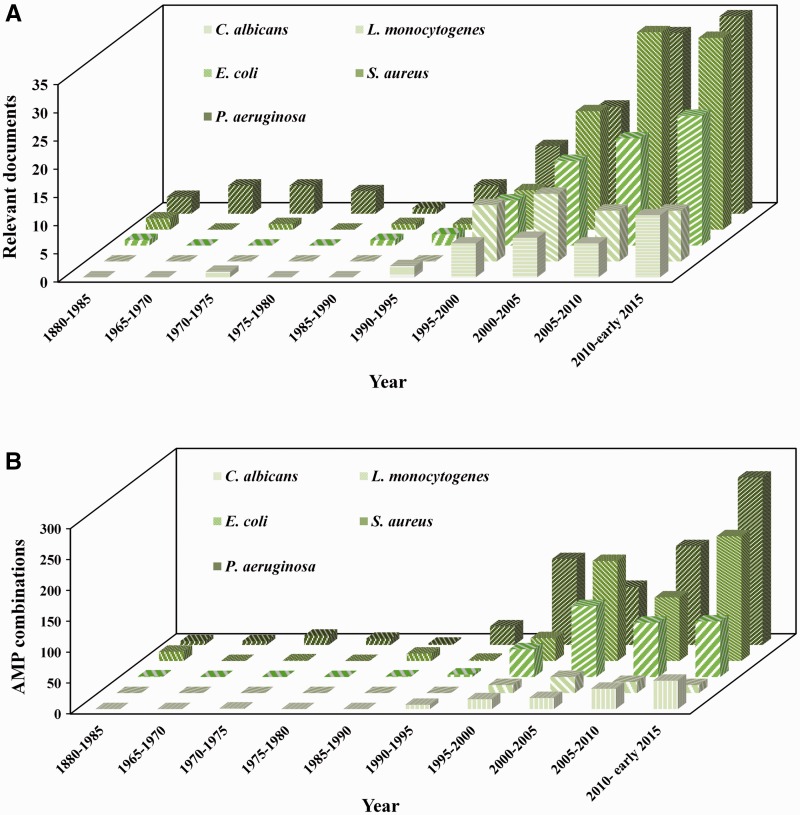
Historical evolution on the publication of AMP-drug combinations. Each row of columns represents data on a different microorganism throughout time, namely **(A)** the number of publications identified as relevant and **(B)** the number of AMP-drug combinations reported.

A retrospective analysis of combination studies may also be interesting to understand existing and prospective lines of research, notably the increasing interest in investigating antimicrobial agents with alternative mechanisms of action. In particular, it is noticeable the attention that AMP are receiving and the growing number of studies testing these agents in combination with conventional compounds and drugs (Figure 2). Most of these studies are devoted to critical pathogenic organisms such as *P. aeruginosa* and *S. aureus*, which have developed severe resistance mechanisms to most of existing antibiotics. Moreover, one may observe that although the number of relevant documents for *P. aeruginosa* and *S. aureus* are not far apart, the number of AMP combinations evaluated is much greater for *P. aeruginosa*. This can indicate that the amount of tested combinations per paper is higher for this bacteria.

### Web interface and user interaction

**Figure 3.**
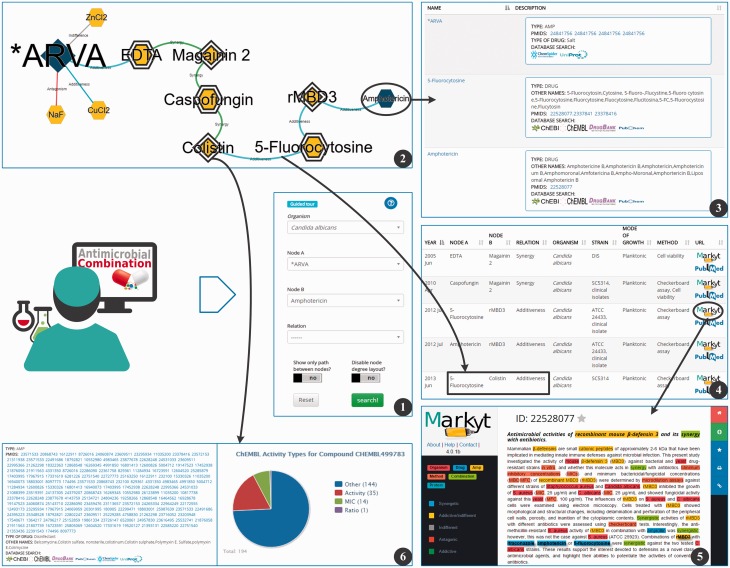
Snapshot of the AMP-drug combination Web search interface. The search page (1) allows for several search criteria. Search results are represented in a network structure (2) that displays the selected nodes (blue coloured), their immediate neighbours and, if applicable, the intermediary nodes that connect the selected nodes. Further details on nodes (3) and edges (4) are provided in additional tables and page views. In particular, the user may always access the available evidences supporting a given antimicrobial combination (5) and all documents referring to a given AMP/drug (6).

The Web interface supporting the reconstructed antimicrobial networks consists of a collection of pages documenting the motivation and goals of the project, and a search functionality to query the curated antimicrobial combinations (Figure 3). The functional view of the network provides several filters to navigate network contents and enables the discovery of indirect associations within the network and among networks.

Users can explore particular antimicrobial combinations or look into all the antimicrobial combinations for one or all organisms. The combinations are displayed as a non-oriented graph. Nodes represent the antimicrobial agents, such that hexagon nodes stand for drugs and circular nodes stand for AMP. Nodes are also coloured according to their network connectivity, i.e. red if the node is connected to <25% of the edges, yellow if the node is connected to 25–50% of the edges, and green if it is connected to >50% of the edges in the database.

Each node provides details on the represented antimicrobial agent, such as alternative names, chemical activity, cross-links to chemical and other external sources. Moreover, the user may access the documents that supported the inclusion of the antimicrobial agent in the network, both the original PubMed record and the curated abstract containing the expert revised annotations. Likewise, each edge describes the nature of the documented combination and available susceptibility data. Once again, the user may access the documents that supported the inclusion of the antimicrobial agent in the graph.

Network visualization is complemented by topological statistics and network details, listed below the graph viewer. Networks can be downloaded as PNG images or in comma separated value format.

### Case study: AMP-drug combination network for *S.* *Aureus*

**Figure 4.**
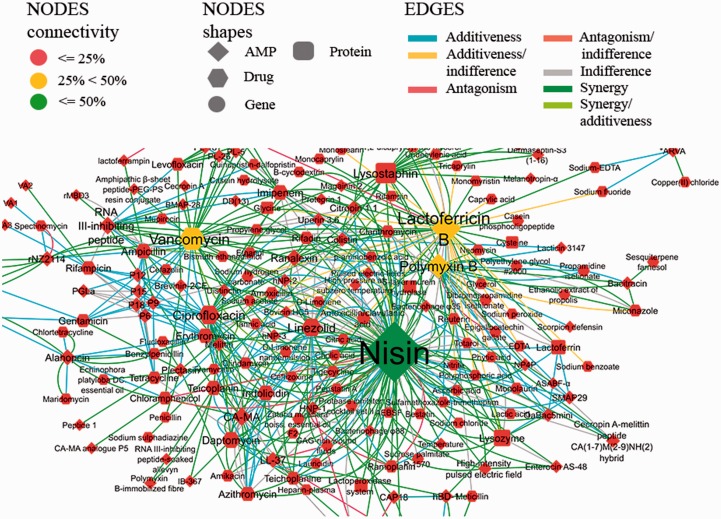
Network showing the AMP combinations tested against *S. aureus*. The size of the nodes correlates directly with its degree of connectivity.

**Figure 5.**
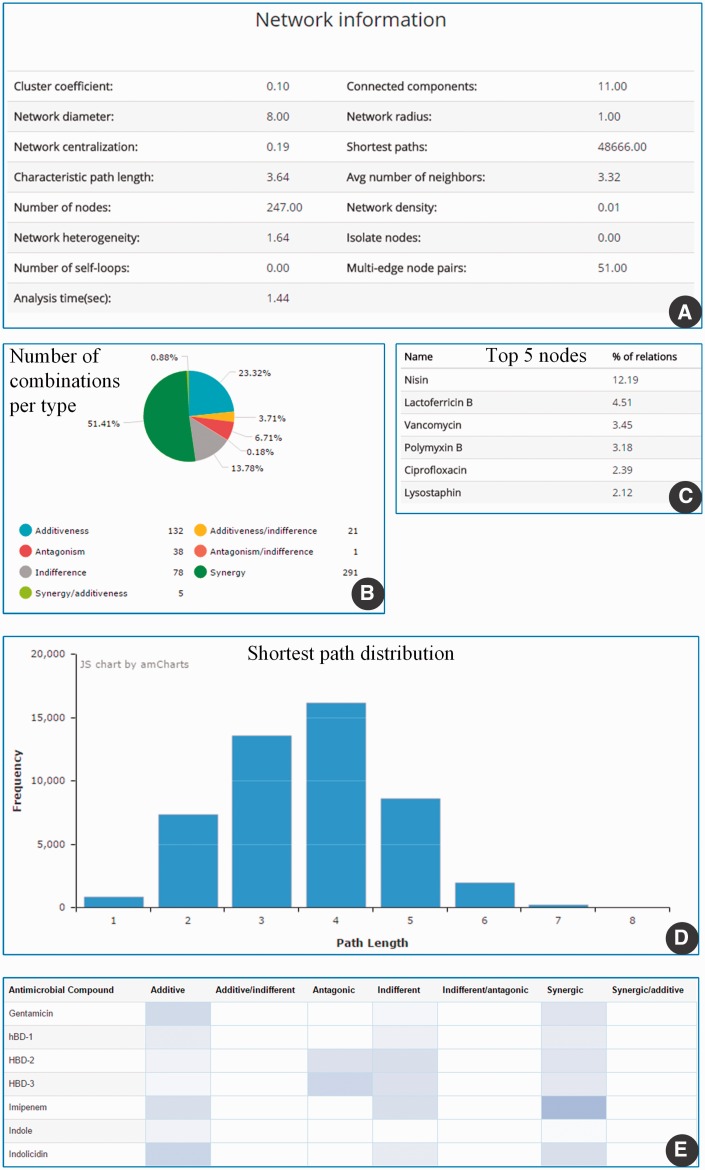
Details on the AMP-drug combinations tested against *S. aureus*. General network statistics **(A)** are detailed in terms of the distribution of combinations by type **(B)**, the identification of the highest degree nodes **(C)**, the distribution of shortest path length **(D)**, and a snapshot of the heat map describing the distribution of all AMP–drug combinations in the database **(E).**

The discussion of the AMP-drug combination network for *S. aureus* is used here as case study to exemplify the exploration of our database. This network contains 224 AMP and drugs and a total of 462 experimentally validated combinations (Figure 4). The network is dominated by a small number of highly connected nodes. In particular, the AMP nisin and lactoferricin B and the antibiotic vancomycin are the most connected nodes (Figures 4 and 5), with degrees of 61, 51 and 35, respectively.

Most of the described combinations for *S. aureus* are of type synergic (>200 combinations) or additive (>100 combinations). The average connectivity of this network is 3.46, i.e. each antimicrobial agent is in average connected to three other antimicrobial agents, and the characteristic path length is ∼4 (Figure 5).

### Discovering and visualizing indirect associations

**Figure 6.**
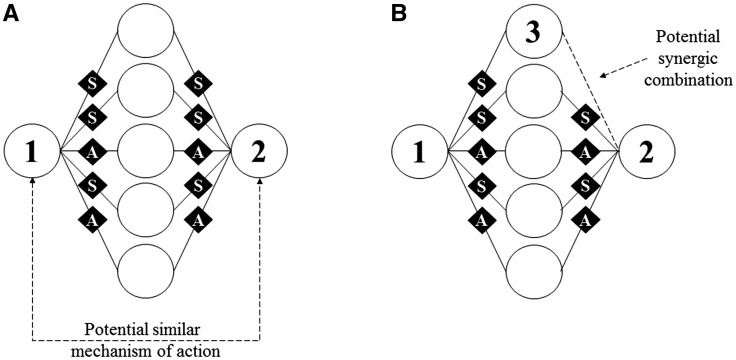
Analysis of indirect associations between antimicrobial agents. **(A)** inference of possible similar mechanisms of action between antimicrobial agents 1 and 2; **(B)** inference of untested promising combination between antimicrobial agents 1 and 3. Circle shapes refer to antimicrobial agents and diamond shapes refer to the type of combinations (A, antagonism; S, synergy).

The term direct association refers to antimicrobial agent combinations that have been experimentally tested and are documented in at least one scientific publication. Conversely, we use the term indirect association to denote two scenarios: the first refers to antimicrobial agents that potentially have the same mechanism of action given their coincident combinations (both in the agents used and the type of combinations) (Figure 6A); the second refers to antimicrobial agent combinations that have not yet been tested but present some potential considering that the individual agents are connected through combinations with other antimicrobial agents (Figure 6B).

The discovery of indirect associations is triggered when the user points two antimicrobial agents. The system displays the sub-graph representing either the direct linking of the two agents, including all the combinations documented for both agents; or, the shortest path between them, including intermediary agents and all the combinations documented for each agents. In both directly and indirectly associated concepts views, users can browse underlying documents by clicking on edges or nodes.

**Figure 7.**
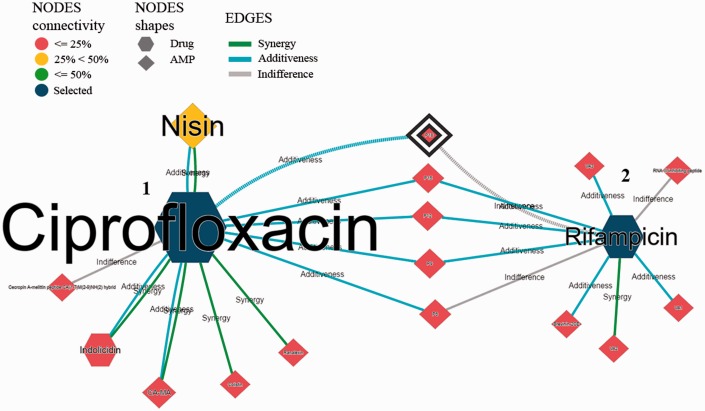
Visualization of indirect associations between ciprofloxacin and rifampicin for *S. aureus* experiments.

Figure 7 shows indirect associations between the antimicrobial agents ciprofloxacin and rifampicin in the *S. aureus* network. The selected drugs are interconnected by 5 ‘intermediary’ agents (the AMP P6, P9, P12, P15 and P18), with no record in the database of the two drugs being tested together. In particular, these 5 combinations of ciprofloxacin with P6, P9, P12, P15 and P18 were documented as additive whereas 3 of these combinations of rifampicin were documented as additive and the other 2 were documented as indifferent (a closely related type of combination to additive).

Both antibiotics have an intracellular action. Notably, their mechanism of action consists in the inhibition of nucleic acid enzymes, with ciprofloxacin inhibiting DNA gyrase and topoisomerase IV (45) and rifampicin inhibiting RNA polymerase (46).Given the similarity of the mechanisms of action of the two drugs and the combinations that they have in common, it is possible to identify promising new combinations (Figure 7). For example, the AMP nisin, CA-MA and indolicidin have between 3 and 6 reported combinations with ciprofloxacin, all with synergic and additive outcomes. So, it could be of interest to test these same AMP with rifampicin in the expectation of obtaining similar positive results (i.e. synergic or additive combinations).

### Discovering and visualizing multiple target combinations

The visualization of AMP combinations across multiple organisms is another supported analysis with multiple applications. Cellular wall and membrane features, which are usually used to divide bacteria into Gram-positive or Gram-negative groups, are known to influence the effectiveness of the antimicrobial agents. Multi-organism visualization for Gram-positive bacteria, e.g. may allow the identification of combinations that are effective across the Gram-positive bacteria analysed and that could be promising candidates for testing in other organisms of the same group.

**Figure 8.**
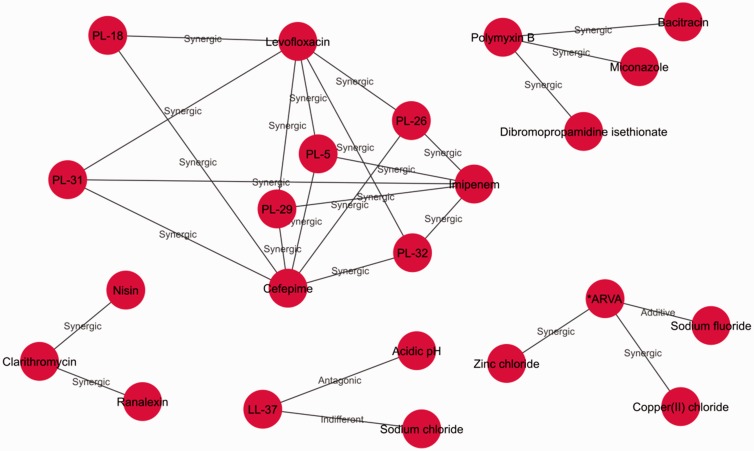
Network of the antimicrobial combinations tested in both *P. aeruginosa* and *S. aureus*. Network intersection was calculated in Cytoscape (48) and accounted for matching antimicrobial combinations (i.e. all antimicrobial combinations experimentally tested for both organisms).

Another possibility is the discovery of effective combinations for two or more organisms that are co-infectious (meaning that they appear together in infections). For example, *P. aeruginosa* and *S. aureus* are found together in many biofilm related infections, such as cystic fibrosis pneumonia, catheter-related infections, diabetic foot ulcers and other wounds (47). The inspection of the intersection network of tested combinations for these two organisms may expand our view of current research (Figure 8). Additionally, by using indirect association analysis (Figure 6) researchers may find combinations, previously untested, and with antimicrobial potential against both pathogens.

## Conclusions

Antimicrobial therapy is being challenged by the ever increasing number of drug resistant organisms, which are rendering most of the conventional drugs unsuccessful. The proposed semi-automated workflow enabled the construction and sustains the update of a new database on experimentally tested antimicrobial agent combinations, with focus on AMP, in order to facilitate the design of more effective antimicrobial treatments. Notably, one of the unique features of our system lies in its capability to identify indirect associations among antimicrobial agents and propose new combinations to be tested.

This workflow integrates semantic analysis of text to identify key information components from biomedical scientific documents, which are then stored in a structured knowledge base over which biomedical queries are processed. Annotation is done in a machine-readable format that allows for the semi-automated curation and display of antimicrobial annotations. The semantic network representation highlights the role of individual antimicrobial agents in various contexts, within and across organisms. Specifically, the query processing module allows users to formulate queries in a guided way at different levels of specificity, such as by organism, antimicrobial agent, and combination effect.

The effort to fully curate new pathogens is considered acceptable. We have a consolidated set of annotation guidelines and the practical and continuous use of semi-automated data pipeline enables the refinement of the automated modules. Most notably, when starting the curation of a new pathogen, experts provide insights into the suitably of the PubMed queries in use and document relevance predictions. Literature about a given pathogen, i.e. the textual contents of the documents, may be sufficiently different to urge for query refactoring and/or model retraining.

In the near future, the analysis of curated combinations for multiple organisms will be extended so that it will be possible to calculate the union, intersection or difference among networks. Likewise, we are investigating the use of deep learning approaches to accelerate manual curation steps. Currently, our workflow is using established methodologies from information retrieval, but deep learning alternatives may be advantageous to improve the generalization ability of the classifier in both local and global scopes.
